# Factors associated with burnout among medical laboratory professionals in Ontario, Canada: An exploratory study during the second wave of the COVID‐19 pandemic

**DOI:** 10.1002/hpm.3460

**Published:** 2022-03-20

**Authors:** Behdin Nowrouzi‐Kia, Jingwen Dong, Basem Gohar, Michelle Hoad

**Affiliations:** ^1^ Department of Occupational Science and Occupational Therapy, Temerty Faculty of Medicine University of Toronto Toronto Ontario Canada; ^2^ Krembil Research Institute‐University Health Network Toronto Ontario Canada; ^3^ Centre for Research in Occupational Safety and Health Laurentian University Sudbury Ontario Canada; ^4^ Shanghai Jiao Tong University Shanghai China; ^5^ Department of Population Medicine The University of Guelph Guelph Ontario Canada; ^6^ Medical Laboratory Professionals Association of Ontario Hamilton Ontario Canada

**Keywords:** burnout, Canada, medical laboratory professionals, medical laboratory technologists, mental health

## Abstract

**Objective:**

The objective of this study was to examine factors associated with burnout among medical laboratory technologists (MLT) in Ontario, Canada during the second wave of coronavirus disease 2019 pandemic.

**Methods:**

We employed a cross‐sectional design and used a self‐reported questionnaire designed for MLT in Ontario, Canada.

**Results:**

There were 441 (47.5% response rate) MLT who were included in the analytic sample. Most of the respondents were women, with a mean age of 43.1 and a standard deviation of 11.7. The prevalence of experiencing burnout was 72.3% for MLT. In the adjusted demographic model, those ≥50 (OR = 0.36, 95% CI: 0.22–0.59) were 0.36 or about one third as likely to experience burnout as those under 50. Similarly, those who held a university degree were less likely to experience burnout compared with high school degree (OR = 0.35, 95% CI: 0.15–0.79). In the adjusted occupational model, high quantitative demands (OR = 2.15, 95% CI: 1.21–3.88), high work pace (OR = 2.21, 95% CI: 1.25–3.98), high job insecurity (OR = 2.56, 95% CI: 1.39–4.82), high work life conflict (OR = 5.08, 95% CI: 2.75–9.64) and high job satisfaction (OR = 0.43, 95% CI: 0.20–0.88), high self‐rated health (OR = 0.32, 95% CI: 0.17–0.56) were significant.

**Conclusion:**

This study provides preliminary evidence regarding the factors associated with burnout in MLT. Additional research is needed to understand their relationship with workers health and well‐being and in the delivery of health services.

## Highlights

- This study examined burnout among medical laboratory technologist in Ontario
- medical laboratory technologists (MLT) experience high rates of burnout during the pandemic
- Demographic factors associated with burnout include education attainment and age
- Occupational factors associated with burnout include high work demands, job demands, low job satisfaction and high self‐regulated health

## 1 INTRODUCTION

Medical laboratory technologists (MLT) are the sixth largest healthcare group in Canada.^1^ In Canada, the primary factors that influence the health of Canadians are the living and working conditions they experience.^2^ Thus influencing the health of MLT working in Ontario. Healthcare is funded by the federal government and delivered by provinces. In Ontario, MLT are regulated healthcare professionals with a restricted title and scope of practice and accountable for their conduct and practice. Medical laboratory technologists work in three distinct areas, including general medical laboratory technology, diagnostic cytology, and clinical genetics.^3^ MLT play a crucial role in the Ontario health system by performing half a million tests each day on blood, body fluids, cells and tissues.^4^ In 2019, there were 20,048 MLT and 7253 MLT who practiced in Ontario.^5^ In Ontario, there has been an increase of 1.78% from the previous year.^5^ However, the number of MLT has decreased −0.47% over the past 4 years.^5^ These healthcare professionals provide critical services that are central to the delivery of healthcare services, yet very little is known about their mental health, including burnout experiences.

The coronavirus disease 2019 (COVID‐19) pandemic continues to place a high demand on healthcare workers who are already highly susceptible to burnout due to the tense work environment and many responsibilities.^6^, ^7^, ^8^, ^9^ Our recent systematic review and meta‐analysis identified nine factors associated (e.g., depression, anxiety, decreased productivity, and burnout) with work performance among healthcare workers including physicians, nurses, and allied health professionals.^10^ The increased workload exacerbates the situation and leads to increased work exhaustion, job dissatisfaction, and turnover rates.^11^ Recent evidence suggests that organisations should consider burnout and develop suitable mitigation strategies specifically for health care professionals including MLT.^12^

Burnout is a syndrome of emotional exhaustion, cynicism, emotional exhaustion, feelings of helplessness, depersonalisation, negative attitude towards work and life, and diminished personal accomplishments.^13^, ^14^ Healthcare professionals are consistently subjected to occupational stress in the delivery of health services, thereby increasing their risk for burnout.^15^ Burnout has been studied extensively in many health care groups, including doctors,^16^, ^17^, ^18^ nurses,^16^, ^19^, ^20^ occupational and physical therapists,^21^, ^22^, ^23^, ^24^ psychologists and social workers.^25^ For example, burnout has been associated with nurses' physical and mental health quality of work life and delivery of patient care.^19^ Studies of occupational therapists^26^ and nurses^27^ reported trial participation predicted work engagement improvements and burnout reductions. Among occupational therapists, focus groups and interviews with the participants resulted in the identification of four primary factors that negatively impact the occupational therapist's day‐to‐day practice: lack of respect (needing to justify decisions/intervention plans); demands on time, lack of autonomy, and conflict (discrepancy between professional values and expectations of the employer).^28^ Similary, physical therapists in Canada have reported a greater risk of demonstrating burnout, but poor response rates hamper conclusions.^21^

Data focussing on burnout in medical laboratory professionals is lacking, particularly in Canada. In general, surveys including but not limited to laboratory professionals, report high levels of burnout.^25^, ^29^ An American cross‐sectional survey of laboratory professionals indicated that 85.3% of respondents experience burnout, and 96.1% experience job stress.^30^ Factors associated with burnout and job stress are understaffing and high workloads. Some studies investigated the factors that can lead to burnout in medical laboratory professionals.^31^, ^32^ A study conducted on factors that lead to work exhaustion in medical technologists reveals that increased levels of perceived work interference with family, increased task load, and lower organizational support were associated with higher work exhaustion.^33^ Another study reported downsizing leads to higher job loss insecurity and increases task load perception, which in turn leads to higher work exhaustion.^34^ Disruptive behaviours in the work environment are also associated with higher rates of burnout.^35^

In Ontario, MLT are experiencing increasing workloads because of the COVID‐19 pandemic leading to deleterious health outcomes including burnout. The objective of this study was to examine factors associated with burnout among MLT in Ontario, Canada.

## 2 METHODS

### 2.1 Design

A cross‐sectional study design was to develop and a questionnaire for MLT in Ontario, Canada. The study is a part of a larger undertaking in Ontario that included 929 MLT and 1866 medical laboratory technicians and assistants and represent approximately 50% of medical laboratory professionals working in Ontario The questionnaire included questions about MLT mental health, well‐being and psychosocial work environments, roles and demographic and occupational characteristics using validated questionnaire. The research project was approved by the research ethics board at the University of Toronto. All participants provided written and electronic consent before taking part in the study. All the data will be collected and securely stored on REDCap^36^ servers at the University of Toronto.

### 2.2 Sample

Medical laboratory technologists were invited with an electronic cover letter stating the study's objectives, description, and respondents' rights as research participants, and a questionnaire. The respondents were provided with two electronic reminders to those MLT who had not responded to the survey. The Medical Laboratory Professionals Association of Ontario (MLPAO) is a provincial organization that represents the interests of medical laboratory professionals with government, health care professionals, regulatory bodies and academic institutions.^37^ The questionnaire and all reminders were distributed electronically by the MLPAO.

All MLT who met the following eligibility criteria were invited to participate in the current study (1) actively registered with the College of MLT of Ontario (only for MLT), (2) Ontario was their clinical practice location, (3) employed and working as of 11 March 2020 (start data of the global pandemic) and (4) position as a MLT providing direct or indirect clinical patient care. In total, 441 MLT met the study's eligibility criteria. We applied a sample size calculation^38^ to determine that this sample size was adequate to detect small to moderate differences in the level of job stress and burnout as perceived MLT working across Ontario, Canada.

### 2.3 Measures

Burnout was examined using the Copenhagen Psychosocial Questionnaire, third edition (COPSOQ‐III)^39^, ^40^ was used to examine the mental health of employees. The questionnaire consists of 48 Likert‐scale questions has 32 psychosocial dimensions (e.g., burnout, stress, job insecurity, quality of work, job satisfaction) across six psychosocial doomains (demands at work, work organization and job contents, interpersonal relations and leadership, work‐individual interface, social capital, health and well‐being).^39^, ^40^ In this study, the burnout symptoms construct was examined as an outcome measure and includes the following two questions “how often have you felt worn out?” and “how often have you been emotionally exhausted?”. The COPSOQ‐III is based on several scales including but not limited to work and emotional demands, burnout, stress, and job satisfaction. The psychometric properties of the COPSOQ‐III were assessed in various countries, including Canada.^39^ Specifically, its reliability (Cronbach α), responses with extreme answers (ceiling and floor effects) and correlations with other dimensions of the COPSOQ‐III (distinctiveness).^39^ As this portion of the study is cross‐sectional, it is unknown if the obtained results are influenced by COVID‐19. To this end, following each domain, the participant selected answered the question “Since COVID‐19, my current response is ___ before” with the following three response options ‘Better than, ‘the same as’, and ‘worse than’. The COPSOQ‐III burnout symptoms also have Canadian standardized data that was used to compare to our population.

### 2.4 Data analysis

Preliminary analyses included tests of the assumptions of the planned inferential statistics. Descriptive statistics were used to characterise the sample regarding its demographic and occupational characteristics. Furthermore, we examined multicollinearity based on a variance inflation factor (VIF) threshold of four (VIF < 5) and models exceeding this threshold were not considered. We used VIF to investigate the severity of multicollinearity in our data.

The impact of the COVID‐19 pandemic on the mental health, and well‐being of MLT was examined using inferential statistics. Speci fically, we used logistic regression to determine the association between demographic (gender, age, marital status, highest level of education attained, ethnicity, number of children living at home) and occupational factors (domains of the COPSOQ III) and burnout. In the occupational model, the independent variables were dichotomised into ‘low’ and high’ by the median values of the distribution as outlined by Gyllensten et al.(2020).^41^ Simiarly, the dependent variable, burnout, was dichotomised into ‘low’ and ‘high’ by the median values of the distributions.^41^ The reference category was identified by the first level of each factor in the model. We used a forward stepwise logistic regression procedure and a significance level for inclusion in the model of *p* < 0.05. The odds ratio and confidence intervals of the logistic regression were reported.

## 3 RESULTS

The characteristics of the study respondents are shown in Table 1. There were 441 MLT with valid results and a response rate of 47.5% (441/929). The prevalence of burnout was 72.3% for MLT (*n* = 319). More than 90% of respondents were women. The mean and standard deviation of the age of the respondents were 43.1 and 11.7, respectively. Overall, nearly half of the workers held a community college degree and 40.8% held a university degree. Respondents were predominantly Caucasian/white, and 54.6% did not have children living at home. Those who required accommodation due to disability accounted for 4.3% for MLT. About three‐quarters of the workers did this laboratory work as a full‐time job.

**TABLE 1:** Demographic and occupational characteristics of medical laboratory technologists (MLT) respondents

	MLT (*n* = 441) %	
Experiencing burnout (*n* = 692)		
Yes	319	72.3
No	122	27.7
Gender (n = 440)		
Male	42	9.6
Female	397	90.2
Other	1	0.2
Age group (n = 415)		
18–35	129	31.1
36–49	116	27.9
50 and over	170	41.0
Marital status (n = 441)		
Single	59	13.4
Married/Common Law/Committed	346	78.5
Separated/Divorced/widowed	36	8.1
Highest level of education attained (n = 441)		
High school	13	3.0
Community college	190	43.1
University	229	51.9
Other	9	2.0
Ethnicity (n = 441)		
Caucasian/White	381	86.4
Other	60	13.6
Number of children living at home (n = 436)		
0	238	54.6
1	56	12.8
2	121	27.8
3	13	3.0
4	8	1.8
5	0	0
Accommodation required at work due to disability (n = 440)		
Yes	19	4.3
No	410	93.2
Prefer not to answer	11	2.5
Employment status (n = 441)		
Full‐time	356	80.7
Part‐time	73	16.4
Other	12	2.7

The impact of the COVID‐19 pandemic was also assessed in the cross‐tabulation of participants and their COPSOQ‐III domain scores. We found that 50.9% (*n* = 224) reported that their job satisfaction was worse than before the start of the pandemic. Furthermore, we found that 77.5% of respondents indicated that experienced higher stress than before the pandemic (Table 2).

**TABLE 2:** Cross‐tabulations of Copenhagen Psychosocial Questionnaire, third edition (COPSOQ‐III) scores and coronavirus disease 2019 (COVID‐19) scores among medical laboratory technologists (MLT)

COPSOQ‐III‐ Domain	COPSOQ score	Since COVID‐19, my response is _____ before			*p* value
Quantitative demands	*N* = 443	Better than *n* = 19 (4.30%)	The same as *n* = 143 (32.3%)	Worse than *n* = 281 (63.4%)	*P* < 0.001
	Low	6	98	94	
	High	13	45	187	
Work pace	*N* = 441	Better than *n* = 15 (3.40%)	The same as *n* = 172 (39.0%)	Worse than *n* = 254 (57.6%)	*P* < 0.001
	Low	8	113	109	
	High	7	59	145	
Emotional demands	*N* = 441	Better than *n* = 4 (0.91%)	The same as *n* = 243 (55.10%)	Worse than *n* = 194 (44.0%)	*P* < 0.001
	Low	1	143	46	
	High	3	100	148	
Influence at work	*N* = 442	Better than *n* = 4 (0.90%)	The same as *n* = 279 (63.1%)	Worse than *n* = 159 (36.0%)	*P* = 0.99
	Low	2	132	76	
	High	2	147	83	
Possibilities for development	*N* = 438	Better than *n* = 21 (4.80%)	The same as *n* = 339 (77.4%)	Worse than *n* = 78 (17.8%)	*P* < 0.01
	Low	6	180	54	
	High	15	159	24	
Meaning of work	*N* = 440	Better than *n* = 65 (14.8%)	The same as *n* = 272 (61.82%)	Worse than *n* = 103 (23.41%)	*P* < 0.001
	Low	17	88	65	
	High	48	184	38	
Predictability	*N* = 439	Better than *n* = 11 (2.50%)	The same as *n* = 213 (48.5%)	Worse than *n* = 215 (49%)	*P* < 0.001
	Low	1	78	125	
	High	10	135	90	
Recognition	*N* = 441	Better than *n* = 24 (5.43%)	The same as *n* = 205 (46.5%)	Worse than *n* = 212 (48.07%)	*P* < 0.001
	Low	1	50	130	
	High	23	155	82	
Role clarity	*N* = 440	Better than *n* = 9 (2.00%)	The same as *n* = 299 (68%)	Worse than *n* = 132 (30.0%)	*P* < 0.001
	Low	5	77	86	
	High	4	222	46	
Role conflict	*N* = 441	Better than *n* = 5 (1.10%)	The same as *n* = 201 (45.6%)	Worse than *n* = 235 (53.3%)	*P* < 0.001
	Low	5	159	110	
	High	0	42	125	
Quality of leadership	*N* = 440	Better than *n* = 18 (4.10%)	The same as *n* = 235 (53.4%)	Worse than *n* = 187 (42.5%)	*P* < 0.001
	Low	1	57	126	
	High	17	178	61	
Social support from colleagues	*N* = 442	Better than *n* = 22 (4.90%)	The same as *n* = 307 (69.5%)	Worse than *n* = 113 (25.6%)	*P* < 0.001
	Low	2	86	78	
	High	20	221	35	
Social support from supervisor	*N* = 440	Better than *n* = 19 (4.32%)	The same as *n* = 271 (61.59%)	Worse than *n* = 150 (34.09%)	*P* < 0.001
	Low	3	122	119	
	High	16	149	31	
Sense of community at Work	*N* = 440	Better than *n* = 21 (4.77%)	The same as *n* = 288 (65.45%)	Worse than *n* = 131 (29.77%)	*P* < 0.001
	Low	0	44	78	
	High	21	244	53	
Insecurity over working conditions	*N* = 439	Better than *n* = 68 (15.5%)	The same as *n* = 285 (64.9%)	Worse than *n* = 86 (19.6%)	*P* < 0.001
	Low	54	187	18	
	High	14	98	68	
Vertical trust	*N* = 441	Better than *n* = 19 (4.30%)	The same as *n* = 259 (58.3%)	Worse than *n* = 163 (37.0%)	*P* < 0.001
	Low	4	63	97	
	High	15	196	66	
Organizational justice	*N* = 440	Better than *n* = 4 (0.90%)	The same as *n* = 242 (55.0%)	Worse than *n* = 194 (44.1%)	*P* < 0.001
	Low	2	105	153	
	High	2	137	41	
Job satisfaction	*N* = 440	Better than *n* = 12 (2.70%)	The same as *n* = 204 (46.4%)	Worse than *n* = 224 (50.9%)	*P* < 0.001
	Low	1	47	140	
	High	11	157	84	
Work life conflict	*N* = 438	Better than *n* = 4 (1.00%)	The same as *n* = 111 (25.34%)	Worse than *n* = 323 (73.74%)	*P* < 0.001
	Low	1	83	102	
	High	3	28	221	
Self‐rated health	*N* = 439	Better than *n* = 8 (1.80%)	The same as *n* = 230 (52.4%)	Worse than *n* = 201 (45.8%)	*P* < 0.001
	Low	2	89	135	
	High	6	141	66	
Burnout	*N* = 440	Better than *n* = 9 (2.00%)	The same as *n* = 95 (21.6%)	Worse than *n* = 336 (76.4%)	*P* < 0.001
	Low	6	57	98	
	High	3	38	238	
Stress	*N* = 440	Better than *n* = 5 (1.10%)	The same as *n* = 94 (21.4%)	Worse than *n* = 341 (77.5%)	*P* < 0.001
	Low	2	73	160	
	High	3	21	181	
Sexual harassment	*N* = 437	Better than *n* = 2 (0.47%)	The same as *n* = 413 (94.5%)	Worse than *n* = 22 (5.03%)	*P* < 0.001
	Low	2	395	15	
	High	0	18	7	
Threat of violence	*N* = 436	Better than *n* = 2 (0.48%)	The same as *n* = 396 (90.8%)	Worse than *n* = 38 (8.72%)	*P* < 0.001
	Low	1	373	19	
	High	1	23	19	
Physical violence	*N* = 436	Better than *n* = 1 (0.23%)	The same as *n* = 410 (94.0%)	Worse than *n* = 25 (5.77%)	*P* < 0.1
	Low	1	398	21	
	High	0	12	4	

The demographic variables associated with burnout are shown in Table 3. The univariate logistic regression analyses revealed that older age (50 and over) was significant factors associated with burnout (OR = 0.44, 95% CI: 0.29–0.68). After controlling for covariates, older age (50 and over) remained significant (OR = 0.36, 95% CI: 0.22–0.59), those who were 50 and over were 0.36 (about one third) as likely to experience burnout as those under 50. Besides, it showed that respondents who held a university degree were less likely to experience burnout compared with high school degree (OR = 0.35, 95% CI: 0.15–0.79).

**TABLE 3:** Multivariable adjusted odds ratio estimates and approximate 95% confidence intervals of demographic and related factors associated with burnout of medical laboratory technologists (MLT)

	Experience burnout at work *n* (%)		Unadjusted odds ratio estimate	95% CI	Adjusted odds ratio estimate	95% CI
	*Yes*	*No*				
Gender						
Male	40 (69.0)	18 (31.0)	1		1	
Female	464 (73.7)	166 (26.3)	1.26	0.70–2.26	0.96	0.49–1.86
Other	2 (67.7)	1 (33.3)				
Age group						
18–35	163 (79.1)	43 (20.9)	1		1	
36–49	171 (77.8)	49 (22.2)	0.92	0.58–1.46	0.82	0.49–1.37
50 and over	142 (62.6)	85 (37.4)	0.44**	0.29–0.68	0.36**	0.22–0.59
Marital status						
Single	83 (72.8)	31 (27.2)	1		1	
Married/Common Law/Committed	387 (74.7)	131 (25.3)	1.10	0.70–1.74	1.17	0.68–2.01
Separated/Divorced/widowed	35 (60.3)	23 (39.7)	0.57	0.29–1.11	0.59	0.27–1.28
Highest level of education attained						
High school	45 (83.3)	9 (16.7)	1		1	
Community college	258 (75.2)	85 (24.8)	0.61	0.29–1.29	0.59	0.26–1.34
University	198 (70.2)	84 (29.8)	0.47	0.22–1.01	0.35**	0.15–0.79
Other	6 (46.2)	7 (53.8)				
Ethnicity						
Caucasian/White	418 (73.7)	149 (26.3)	1		1	
Other	89 (71.2)	36 (28.8)	0.88	0.57–1.36	0.78	0.48–1.28
Number of children living at home						
0	259 (72.3)	99 (27.7)	1		1	
1	70 (74.5)	24 (25.5)	1.12	0.66–1.87	1.08	0.60–1.94
2	136 (76.4)	42 (23.6)	1.24	0.82–1.88	1.20	0.75–1.93
3	23 (69.7)	10 (30.3)	0.88	0.40–1.91	0.76	0.32–1.77
4	10 (76.9)	3 (23.1)	1.27	0.34–4.73	1.23	0.32–4.73
5	1 (50.0)	1 (50.0)				

*Note:* *p < 0.05,**p < 0.01.

The relationships between occupational and workplace psychosocial characteristics and burnout are shown in Table 4. Univariate logistic regression analyses showed that 23 of the 25 psychosocial domains (except influence at work and sexual harassment) were significantly associated with experiencing burnout. In the stepwise multivariate logistic regression analyses, of the 23 significant variables, high quantitative demands (OR = 2.15, 95% CI: 1.21–3.88), high work pace (OR = 2.21, 95% CI: 1.25–3.98), high job insecurity (OR = 2.56, 95% CI: 1.39–4.82), high work life conflict (OR = 5.08, 95% CI: 2.75–9.64) and high job satisfaction (OR = 0.43, 95% CI: 0.20–0.88), high self‐rated health (OR = 0.32, 95% CI: 0.17–0.56) remained significant. In other words, for example, those who experienced high quantitative demands at work had a 2.15 times higher risk of burnout compared to those who had experienced low quantitative demands at work.

**TABLE 4:** Multivariable adjusted odds ratio estimates and approximate 95% confidence intervals of job and career satisfaction factors associated with burnout of medical laboratory technologists (MLT)

	Experience burnout at work *n* (%)		Unadjusted odds ratio estimate	95% CI	Adjusted odds ratio estimate	95% CI
	*Yes*	*No*				
Employment status						
Full‐time	393 (75.0)	131 (25.0)	1		1	
Part‐time	104 (71.7)	41 (28.3)	0.85	0.56–1.29	0.87	0.45–1.67
Other	10 (43.5)	13 (56.5)				
Quantitative demands						
Low	181 (57.8)	132 (42.2)	1		1	
High	324 (86.6)	50 (13.4)	4.73**	3.27–6.91	2.15**	1.21–3.88
Work pace						
Low	198 (60.0)	132 (40.0)	1		1	
High	307 (86.7)	47 (13.3)	4.36**	3.00–6.40	2.21**	1.25–3.98
Emotional demands						
Low	231 (60.9)	148 (39.1)	1		1	
High	273 (89.5)	32 (10.5)	5.47**	3.63–8.44	1.71	0.88–3.36
Influence at work						
Low	267 (76.9)	80 (23.1)	1		1	
High	238 (70.6)	99 (29.4)	0.72	0.51–1.01	0.57	0.31–1.07
Possibilities for development						
Low	306 (76.7)	93 (23.3)	1		1	
High	198 (69.0)	89 (31.0)	0.68**	0.48–0.95	0.90	0.48–1.67
Meaning of work						
Low	222 (79.9)	56 (20.1)	1		1	
High	282 (69.6)	123 (30.4)	0.58**	0.40–0.83	0.81	0.44–1.50
Predictability						
Low	268 (85.1)	47 (14.9)	1		1	
High	237 (63.9)	134 (36.1)	0.31**	0.21–0.45	1.13	0.59–2.18
Recognition						
Low	257 (87.4)	37 (12.6)	1		1	
High	248 (63.3)	144 (36.7)	0.25**	0.16–0.37	1.41	0.65–3.06
Role clarity						
Low	238 (88.8)	30 (10.2)	1		1	
High	266 (63.8)	151 (36.2)	0.22**	0.14–0.34	1.08	0.51–2.31
Role conflicts						
Low	251 (63.9)	142 (36.1)	1		1	
High	254 (86.7)	39 (13.3)	3.69**	2.50–5.53	0.85	0.42–1.70
Quality of leadership						
Low	231 (83.4)	46 (16.6)	1		1	
High	272 (67.2)	133 (32.8)	0.41**	0.28–0.59	1.24	0.58–2.71
Social support from colleagues						
Low	237 (86.2)	38 (13.8)	1		1	
High	268 (65.2)	143 (34.8)	0.30**	0.20–0.44	0.58	0.29–1.14
Social support from supervisor						
Low	312 (81.7)	70 (18.3)	1		1	
High	193 (63.7)	110 (36.3)	0.39**	0.28–0.56	0.71	0.37–1.38
Sense of community at work						
Low	184 (86.4)	29 (13.6)	1		1	
High	320 (67.7)	153 (32.3)	0.33**	0.21–0.50	1.28	0.62–2.67
Job insecurity						
Low	309 (69.8)	134 (30.2)	1		1	
High	194 (80.8)	46 (19.2)	1.83**	1.26–2.70	2.56**	1.39–4.82
Insecurity over working conditions						
Low	257 (69.5)	113 (30.5)	1		1	
High	248 (78.2)	69 (21.8)	1.58**	1.12–2.24	1.12	0.63–2.00
Vertical trust						
Low	220 (88.0)	30 (12.0)	1		1	
High	284 (65.3)	151 (34.7)	0.26**	0.16–0.39	0.61	0.29–1.28
Organizational justice						
Low	225 (89.3)	27 (10.7)	1		1	
High	278 (64.2)	155 (35.8)	0.22**	0.14–0.33	0.50	0.23–1.06
Job satisfaction						
Low	281 (92.7)	22 (7.3)	1		1	
High	225 (58.4)	160 (41.6)	0.11**	0.07–0.17	0.43**	0.20–0.88
Work life conflict						
Low	140 (48.4)	149 (51.6)	1		1	
High	366 (92.7)	29 (7.3)	13.43**	8.75–21.26	5.08**	2.75–9.64
Self‐rated health						
Low	317 (88.3)	42 (11.7)	1		1	
High	189 (57.3)	141 (42.7)	0.18**	0.12–0.26	0.32**	0.17–0.56
Sexual harassment						
Low	459 (73.2)	168 (26.8)	1		1	
High	48 (80.0)	12 (20.0)	1.46	0.78–2.95	0.49	0.16–1.45
Threats of violence						
Low	392 (70.8)	162 (29.2)	1		1	
High	114 (85.7)	19 (14.3)	2.48**	1.51–4.28	0.96	0.34–2.79
Physical violence						
Low	446 (72.3)	171 (27.7)	1		1	
High	59 (84.3)	11 (15.7)	2.06**	1.10–4.22	2.38	0.69–8.73
Bullying						
Low	261 (68.5)	120 (31.5)	1		1	
High	246 (80.1)	61 (19.9)	1.85**	1.31–2.65	0.81	0.44–1.49

*Note:* ¥ *p < 0.05,**p < 0.01.

## 4 DISCUSSION

The objective of this study was factors associated with burnout among MLT. Specifially, this study investigated the relationship between demographic and occupational factors and burnout among MLT groups in Canada. In the adjusted demographics model, we found respondents ≥50 (OR = 0.36, 95% CI: 0.22–0.59) were about one third as likely to experience burnout as those under 50. Participants with a university degree were less likely to experience burnout compared with high school degree (OR = 0.35, 95% CI: 0.15–0.79). In the adjusted occupational model, high quantitative demands (OR = 2.15, 95% CI: 1.21–3.88), high work pace (OR = 2.21, 95% CI: 1.25–3.98), high job insecurity (OR = 2.56, 95% CI: 1.39–4.82), high work life conflict (OR = 5.08, 95% CI: 2.75–9.64) and high job satisfaction (OR = 0.43, 95% CI: 0.20–0.88), high self‐rated health (OR = 0.32, 95% CI: 0.17–0.56) were significant. The overall prevalence of burnout in medical laboratory professionals was 73.3%, higher than most other healthcare workers during COVID‐19,^42^, ^43^, ^44^ like doctors, occupational therapists, nurses, and pharmacists. This conclusion was also reported in a Malaysian mixed‐method study,^25^ sugesting decreased investment in the health system, higher workload, and the lack of new equipment. There were several reasons for the phenomenon that MLT experienced burnout relatively frequently. A study mentioned that the leading causes of burnout among MLT may be inadequate staffing and pressure to complete all testing.^45^ Another Japanese study suggested that this might also contribute to the idea that nonphysicians could be that these job categories have lower dimensions of control (skill discretion and decision authority) compared with physicians.^46^

The relationships between psychosocial risk factors and burnout that we found were similar to those reported in earlier studies. For example, Freimann et al.^47^ who also used the COPSOQ questionnaire, found that quantitative demands (workload), emotional demands, work pace and role conflicts had a significantly positive correlation with stress psychosocial risk factors studied significantly correlated with burnout. In our research, high quantitative demands, work pace, job insecurity and work life conflict positively associated with burnout. Laboratory professionals in such circumstances (high quantitative demands) were experiencing a lot of pressure at their workplace, because they did not have enough time to complete the tasks and felt insecure, despite giving maximum effort. Job satisfaction and high self‐rated health represented protective psychosocial factors. This finding was in line with the study in Belgian^48^ and Hungarian.^49^ These results drew our attention to the importance of improving the psychosocial work environment among MLT.

The multivariate logistic regression analysis revealed that older age group and lower education level were significantly associated with burnout. These findings are generally in agreement with the literature. In many studies, lower education levels are the primary risk factors for experiencing burnout.^50^, ^51^ However, a study showed that those with better education were less satisfied with their jobs.^51^ As mentioned in the last paragraph, job satisfaction was a protective predictor of burnout. This seemed to be a paradoxical conclusion, as we explained that the anti‐burnout experience of a high degree outweighed the depression associated with low job satisfaction.

### 4.1 Strengths and limitations

This is the first study of its kind to examine the psychosocial work environment of MLT and factors associated with burnout among medical laboratory professionals. Moreover, collecting data during the COVID‐19 pandemic will allow for comparing the findings with future studies. These health professionals serve as the backbone of the health care system as they provide testing results and serving on the frontline of the COVID‐19 pandemic. However, their mental health is poorly understood and not well explored. The main strengths of the present study were that: (a) the participants were from Medical MLPAO, which was an organization that supported MLTs by representing their interest with government, regulatory bodies, educational institutions, health care professions and other stakeholders. It should be recognized that our study results may be useful to policymakers and the public, who tend to consider a broad range of occupational factors in addition to demographic characteristics. (b) using internationally well‐known measurements, we can interpret our findings considering international data.

We also acknowledge other potential limitations. First, the study is cross‐sectional; thus, no causal relationships can be made; Second, the low completion rate of the questionnaire may lower the generalisability of results. Third, there are different versions of COPSOQ‐III that may raise questions about the validity and reliability of the results.

## 5 CONCLUSION

Burnout is a significant health concern for medical laboratory professionals worldwide. We hope to expand our sample and work with other universities or government organisations to collect data from the international perspective to understand the impacts of the COVID‐19 pandemic on medical laboratory professionals.

The findings could also help us develop future interventions in medical laboratory professionals. Healthcare organisations may utilise the findings to develop policies for pandemic planning, programs, services and practices designed specifically for a public health crisis such as the COVID‐19 pandemic. As a result, we are planning to have virtual interviews with MLT to discuss the impact of COVID‐19 on mental health within the context of work and help learn more about coping methods.

Our study warrants further investigations using larger sample sizes and the development of interventions to support medical laboratory professionals' mental health and well‐being. All stakeholders, including governments, hospitals, public and private clinics, must work closely with these healthcare professionals to address their mental health and ensure a work environment where their health and safety is embedded into its culture.

## ETHICS STATEMENT

The study was approved by the University of Toronto Research Ethics Board.

## Data Availability

The data that support the findings of this study are available from the corresponding author upon reasonable request.
